# Fetal Origin of Sensorimotor Behavior

**DOI:** 10.3389/fnbot.2018.00023

**Published:** 2018-05-23

**Authors:** Jaqueline Fagard, Rana Esseily, Lisa Jacquey, Kevin O’Regan, Eszter Somogyi

**Affiliations:** ^1^Laboratoire Psychologie de la Perception (UMR 8242), CNRS—Université Paris Descartes, Paris, France; ^2^EA 3456—Laboratoire Éthologie Cognition Développement (LECD), Université Paris Ouest Nanterre, Nanterre, France

**Keywords:** fetus, precursor, sensorimotor development, motor babbling, mechanisms

## Abstract

The aim of this article is to track the fetal origin of infants’ sensorimotor behavior. We consider development as the self-organizing emergence of complex forms from spontaneously generated activity, governed by the innate capacity to detect and memorize the consequences of spontaneous activity (contingencies), and constrained by the sensory and motor maturation of the body. In support of this view, we show how observations on fetuses and also several fetal experiments suggest that the fetus’s first motor activity allows it to feel the space around it and to feel its body and the consequences of its movements on its body. This primitive motor babbling gives way progressively to sensorimotor behavior which already possesses most of the characteristics of infants’ later behavior: repetition of actions leading to sensations, intentionality, some motor control and oriented reactions to sensory stimulation. In this way the fetus can start developing a body map and acquiring knowledge of its limited physical and social environment.

## Fetal Development

### The Very Beginning of Motility…

Spontaneous motoneuron activity begins at the same time as motoneuron differentiation. Indeed, motor activity starts as rhythmic bursts of spontaneously generated action potentials correlated across thousands of cells, at a stage when motor neurons are pathfinding and innervating the skeletal muscles. Among other cellular processes, these periodic bursts of action potentials increase the concentration of calcium in the neurons, influencing gene expression and the establishment of cell phenotype (Feller, 1999; Kirkby et al., 2013). More generally, correlated neuronal activity guides neuronal differentiation, migration, synaptogenesis and development of neuronal networks (Milh et al., 2007). Initiated by stochastic bursts in the spinal cord and brainstem networks, the first noticeable movements occur after the 6th week of gestation age. At $7-7\frac{1}{2}$ weeks of gestation, sideways bending of the head or of the rump can be observed with ultrasound recording. These movements, simple and stereotyped, soon disappear (Kurjak et al., 2002, 2004; Hadders-Algra, 2007).

Motor patterns most characteristic of the first weeks of gestation are spontaneous startles, general movements (GMs), isolated movements and twitches^1^. Around 7–8 weeks of age, the fetus makes occasional startles. Startles increase in frequency until they reach a peak, followed by a strong decrease up to 17 weeks and a slower decrease till the end of pregnancy^2^. At the beginning, startles are often followed by GMs, in which all parts of the body participate. The first GMs, which appear at about 8 weeks (de Vries et al., 1985; Kurjak et al., 2008) are always preceded by a startle (Piontelli, 2010). It is possible that the massive displacements due to startles, which trigger a chain of counter-reactive movements, facilitate the initiation of GMs (Piontelli, 2010; however, de Vries et al., 1985 consider that there is no relationship between the occurrence of startles and GMs). GMs are characterized by their fluency and their variety, and their repertoire increases rapidly from 8 weeks to 10 weeks (de Vries et al., 1985; Lüchinger et al., 2008). The emergence of GMs happens at the same age at which the motoneurons of the spinal cord are connected to the subplate, a transient brain structure underneath the cortical plate before it develops (de Graaf-Peters and Hadders-Algra, 2006). After the 17th week, GMs do not necessarily follow a startle and appear spontaneously.

As opposed to GMs, isolated movements, which emerge soon after GMs and outnumber them by the 14th week, involve distinctive sequencing of particular body parts (Prechtl, 1990; Roodenburg et al., 1991; Hadders-Algra, 2007; Kurjak et al., 2008). The onset of isolated movements is simultaneous for arms and legs (Kurjak et al., 2008) but arm movements are more frequent than leg movements, at least in 14- to 18-week fetuses (Kuno et al., 2001). The incidence of isolated arm movements increases gradually from 8 through 19 weeks, which is in contrast with isolated leg movements which practically do not increase and even decrease after 15 weeks (de Vries et al., 1985).

Twitches are a particular kind of spontaneous motor activity produced during active sleep. Brief contractions of muscles trigger quick extensions or flexions of a limb or the neck. Fetuses start producing twitches at the age of 10–12 weeks, and from 15–16 weeks the frequency of twitches increases substantially. As we will see later, even though they appear during sleep (and according to some authors possibly because they appear during sleep) twitches may have an important role for establishing the brain’s body map (Blumberg et al., 2013).

In general, the number of fetal movements per hour increases until a plateau is reached and decreases from 16 weeks onward (Natale et al., 1985; Roodenburg et al., 1991). The periods of quiescence (without GMs, isolated movements of all sorts including limb movements, trunk movements, head movements, mouth movements (jaw opening, yawning), hiccups, facial movements, etc.) are very short until 20 weeks (13 min maximum; de Vries et al., 1985).

### …Immediately Followed by Sensory Experience and Sensorimotor Behavior

Even though early motility appears to be mostly unrelated to sensations, it is difficult to determine precisely when a fetal movement is spontaneously initiated or when it is triggered by sensations, due to movements of the mother or to internal sensations. Reflexive reactions to touch occur almost as early as spontaneous motor behavior. They are first observed in the region around the mouth: for instance, after stroking the perioral region, contraction of the neck muscles on the side opposite the stimulation, making the surface touched move away from the stimulator, has been observed at 7–8 weeks (Hooker, 1952, cited in Hadders-Algra, 2007). Early instances of reaction to “ecological” touch can be observed in the case of twins: twins react strongly when they are touched or pushed by the other twin. Such reactions can be observed between 11 and 13 weeks—earlier in the case of monochorionic twins (Piontelli, 2010).

As the sensory systems develop, non-reflexive responses to stimulations can be observed. The fetus’ environment is often disturbed by sounds, light, and touch and the fetus soon responds to these disturbances by moving (Valman and Pearson, 1980). Experiments show that the fetus not only responds to external stimulations but orients toward their source (or away; Lecanuet et al., 1989). Twenty-one to thirty-three-week-old fetuses respond to maternal touch of the abdomen or to vibroacoustic probes by an increase of arm, head and mouth movements (Marx and Nagy, 2015) and also by changes in fetal heart rate. Fetuses respond first to tactile, and then vibroacoustic, auditory and visual stimulations, but olfactory sensations are also processed (Schaal and Orgeur, 1992; for a review see Kisilevsky and Low, 1998). Many fetal movements, however, are clearly spontaneously triggered and are not a response to stimulation. Fetuses do a lot of “motor babbling.”

### Motor Babbling (GMs and Isolated Movements)

The first movements of the fetus, general or isolated, give the impression, not only of being spontaneous and not in reaction to sensation, but also of not being aimed at a precise goal, but rather to be randomly distributed across the space around it. We refer to motor babbling when movements seem random (Caligiore et al., 2008). Even in the apparent absence of an intentional goal, these movements allow the fetus to explore the space around it, to explore its body and its environment and to explore the consequences of its movements on its body and on its environment. One could assimilate fetuses to astronauts exploring space, driven by some kind of primitive curiosity or intrinsic motivation. Intrinsic motivation refers to living creatures’ (especially young ones’) search for novelty, in other words to a behavior that does not lead to the satisfaction of physiological needs but rather to an increase of knowledge (about own body and environment).

Movements are not explored equally by the fetus. Early fetal movements are canalized by some constraints, arising from the system itself (characteristics of the articulations, state of development of the nervous system) as well as by the characteristics of the environment. These characteristics change through pregnancy, and due to these changes the contingent effect of the same movements may change. Due to the aquatic environment, arm and leg movements are likely to turn the body around as long as there is enough space and enough amniotic fluid around the fetus. By the end of pregnancy, when space is shrinking as the fetus grows, most arm movements end up not far from the face. The nervous system also is changing. From the beginning, there are two cortico-spinal tracks, one descending directly toward the spinal cord and the periphery (ipsilateral), the other one crossing the corpus callosum and descending on the opposite side of the spinal cord and periphery (contralateral). At first control is ipsilateral but becomes increasingly contralateral as the corpus callosum develops (Malinger and Zakut, 1993). The decrease in the amount of movement during pregnancy is believed to be due, not only to the restriction in available space, but also to the emergence of inhibitory cortical influences.

Within these constraints, babbling is extremely variable within and across fetuses. It may result in accidental contacts with the body or with the uterine environment. Such accidental contacts appear to be held in a memory of consequences, in such a way that the fetus soon starts to show a repertoire of “preferred” movements, as we will see in the next paragraph.

### From Motor Babbling to Sensorimotor Map

To repeat a movement, the fetus must know the connections between motoneurons and muscles, in other words it must have some sort of sensorimotor mapping. Scientists increasingly believe that sensorimotor mapping emerges progressively from spontaneous movements. Indeed, there are no movements without sensory consequences (the reverse being not true since sensory stimulations are not always followed by movements). Even twitches, produced on a background of muscle atonia (during sleep), are believed to play a fundamental role in the self-organization of spinal and supraspinal sensorimotor circuits and body mapping (Blumberg et al., 2013, 11393). GMs are like sensorimotor “storms” during which tactile, proprioceptive and vestibular sensations are simultaneously elicited (Piontelli, 2010). Isolated movements allow the fetus to touch different parts of the body, with the back or with the palm of the hand, or with the foot. Touching induces double tactile stimulation: stimulation of the active touching part (hand, leg, tongue) and passive stimulation of the touched part. Therefore, even before the brain starts to receive significant sensory input from the outside world, spontaneous movements provide sensory stimulations. As a result, GMs and isolated movements are important not only, as all other spontaneous movements, for the development of the motor machinery of muscles, tendons, ligaments, cartilages, spindles and bones (Müller, 2003), but also for the development of sensorimotor circuits and sensorimotor mapping.

Thus, fetal sensory stimulations arise from several sources, from endogenously-triggered spontaneous movements as well as from other sources, to stimulation arising from fetal or outside environment. All are likely to contribute to the development of somatosensory cortex and to the formation of cortical body maps (Milh et al., 2007).

### Early Goal-Directed Movements, Channeled by Sensory Consequences

With isolated movements, fetuses soon seem to increasingly prefer those parts that are richly innervated. Starting at 10–12 weeks, face contacts are seen very often, which is interesting knowing that the trigeminal, which innervates the face, is an important source of tactile and proprioceptive sensations (Kurjak et al., 2008). Arm movements aim increasingly toward the mouth as pregnancy progresses (Piontelli, 2010). The mouth and the thumb are both highly innervated and we hypothesize that this is the reason why fetuses seem to like thumb sucking (see also Piontelli, 2010). Other self-touch behaviors observed *in utero* include rubbing the eyelids, scratching the temples with the fingers, which, even without nails, may elicit sensations. The cranium, which is very little innervated by sensory fibers, is rarely scratched, except the part that is more innervated such as the occiput and the nape. After the first eye motions, at 16–18 weeks, the fetus starts touching its eyelids, closed until 23–24 weeks. Retina development is well advanced at that age and rubbing the eyelids may generate flashes of light in the fetus. Fetuses also touch their feet, in particular the soles that are well innervated. Fetuses rarely touch other parts of their body that are less sensitive, like the abdomen or the thorax. The most frequent movements in the third trimester are facial movements (eyelids and mouthing movements; hand to face, hand to eye, hand to head, scowling, eye and mouth opening, Kurjak et al., 2004). Comparing 21–26-week-old fetuses with 26–33-week-old Marx and Nagy (2015) observed more self-touch behaviors in the older group.

At first, hands move independently. At 20–22 weeks, fetuses can be seen touching one hand with the other or crossing hands. They may also grasp the umbilical cord when they accidently contact it, thanks to the grasping reflex (Piontelli, 2010).

If the fetus increasingly aims its movements toward the more sensitive body parts, this means that it progressively selects these movements that induce interesting sensory feedback. Indeed, recent observations suggest that the fetus is capable of anticipating the consequences of its movements, which may be a first step toward action planning. For instance, two studies showed that fetuses anticipate their movement toward the mouth by opening the mouth before the hand arrives (Myowa-Yamakoshi and Takeshita, 2006; Reissland et al., 2014). This anticipation seems to increase in frequency as gestation progresses. Another study showed that the arm movements toward the mouth become more direct from the 22nd week onwards than before. In addition, the dynamics of arm movements heading toward the mouth is different from the dynamics of the movements toward the eye, for which the arm slows earlier during the movement, reaching the eye more carefully than the mouth (Zoia et al., 2007). It is around the same age (25–34 weeks) that the cortical plate becomes organized in six layers and that axons reach the cortex.

### Social Aspect of Sensorimotor Movements

Fetal behavior already presents some social characteristics observed in neonates. We have already mentioned the specific response to the mother’s touching her abdomen (Marx and Nagy, 2015). In the same study, the authors showed that the fetus also responds specifically to the mother’s voice. Other recent studies indicate social responsiveness of the fetus during the third trimester. In one study with 25-week-old fetuses, the authors observed more mouth opening immediately followed by closing when the mother sang the syllable “LA” in a nursery rhyme than in any other stimulation (chewing, yawning, etc.; Ferrari et al., 2016). In a similar study, fetuses reacted with a specific configuration of mouth opening to hearing the sound “ma” being repeated (Reissland et al., 2016). Finally, in another study which has still to be replicated, 25-week-old fetuses were more likely to engage with upright face-like visual stimulus presented through the uterus than with inverted ones^3^ (Reid et al., 2017). Although an interpretation in terms of innate knowledge could be tempting from a nativist point of view, it could also be that the fetus’s frequent explorations of its eyes and mouth give it more familiarity with one configuration than with the other. One would have to consider that cross-modality exists in the fetus: since cross-modality can be observed in newborns, as we will see further, there is no reason to exclude that it exists already at the late fetal stage.

In conclusion, the fetus’ motility is no longer seen as a purely reflexive behavior, or as simply emerging from motor primitives hardwired in the spinal cord or brainstem. And development itself is no longer considered as the results of increasing cortical control over lower reflexes through an unfolding program. Rather, development is now considered as the self-organizing emergence of complex forms from the spontaneously generated activity inherent in individuals with a nervous system, from the sensory constraints due, for instance, to the non-uniform distribution of tactile sensors, and from the capacity to detect and memorize the consequences of spontaneous activity (see, for instance Yamada et al., 2010; or Gottlieb’s developmental system view and canalization at several levels of the developing system, from genes and system nervous to experience in the environment, Gottlieb, 1991; Hadders-Algra, 2007). It is this interaction between genetically-driven spontaneous activity, genetically-driven basic capacities to detect affordances and regularities, and constraints or channeling due to body and environment that explains behavioral development. Moving and its sensory consequences allows the fetus to pick up information for making sense of itself and the world, in other words allows sampling of itself and of the world through action. In turn, this continuous sampling of information modifies input statistics. This leads to changes in brain networks, permitting new behaviors (for a dynamic model of how brain networks and behavior in the environment’s continuous reciprocal interactions accounts for changes in development, see Byrge et al., 2014).

As isolated movements change along pregnancy, the fetus’ sensorimotor behavior comes to possess some of the characteristics later observed in the child’s behavior: curiosity or intrinsic motivation to explore surrounding space and the body, detection of contingencies, repetition of actions leading to sensations, reaction to sensory inputs, intentionality, goal-directed movements and some motor control. It is noteworthy that fetuses already display habituation, namely the decrease in reaction as a repeated stimulus loses its novelty, with vibroacoustic stimulation, speech sequences, and tones: such habituation has been observed in fetal heart changes and body movements (Lecanuet et al., 1989, 1992; Kisilevsky and Low, 1998) as well as using brain imaging (30–39 weeks, fMEG, Muenssinger et al., 2013). The difficulty in considering sensorimotor fetal behavior as already possessing the main characteristics as infants’ sensorimotor behavior is that birth may create a discontinuity in motor control and that one has to wait a few weeks to observe the infants displaying a behavior comparable to the fetal behavior.

## Birth: Rupture and Continuity

### First Weeks of Life

There is some continuity between fetal and neonate motor behavior in the sense that all movements observed in fetal life are present in neonatal life (Kurjak et al., 2004). The constraints due to the neural system do not change at birth. In contrast, environmental constraints do change tremendously: the neonate goes from an aquatic to an aerial medium, from an almost dark environment to a bright one. This may explain the apparent regression in motor control as the newborn has many new parameters to integrate into its movements. The neonate is able to control its ocular system rather well (Farroni et al., 2004). In special conditions, it may approach a visual target with its hands (Bower et al., 1970; Grenier, 1981; von Hofsten, 1982). In addition, it can detect contingencies and try to reproduce them: it accelerates its rhythm of sucking if this allows it to hear its mother’s voice (DeCasper and Prescott, 2009); it slightly raises its arm to see the arm in a beam of light (van der Meer, 1997). However, it will take a few weeks to see the infant playing regularly with its body, and more to see it spontaneously reaching for an object.

During the first 2 months the infant adapts to a new environment, new feeding, rhythm of day and night, it frequently has digestive problems, so that even though it has moments of clear awakening and interaction with people, most of its time is shared between feeding, crying, sleeping. This leaves little time for exploring the world, including itself. The emotional reactions of the baby interacting with its social partners are the most significant behaviors at that age. Then, around 2 or 3 months, infants are seen exploring their own body frequently.

### Exploring Own Body: Self-Touch Behaviors

Self-touch re-appears shortly after birth with little variations from right before birth, except for hand to mouth which increases and hand to knee which decreases (Sparling et al., 1999) and one of its functions is believed to be self-soothing (Durier et al., 2015). A recent analysis of movements in relation to body area touched, of 42 resting alert infants seen biweekly from birth to 6 months, has shown a developmental tendency in self-touching movements (Thomas et al., 2015). At first, infants mainly made contact with the head, torso, arm and hands. At 12 weeks contacts with hips and upper thighs became frequent, and finally after 20 weeks, infants often contacted their knees and feet after bending the knees and bringing the feet up to the torso. The authors also observed that the younger infants often touch their body with the dorsal part of the hand, that palmar contacts increase with age, and that by 16 weeks of age the proportion of grasp contacts increases. Grasp contacts consist in closing of one or more of the digits or the whole hand around the infant’s body or clothing. Thus, the developmental trend in self-touching includes a tendency to go from rostral to caudal targets, from dorsal to palmar hand contacts, and from touch to grasp behaviors (see also Wallace and Whishaw, 2003). This rostro-caudal tendency has also been observed for detecting external stimulation on the body: by 3 months of age infants bring one hand into contact with the other when a buzzer is applied to the latter. The same sensory input on one segment of the body appears to be detected later for the feet (Somogyi et al., 2017).

### First Object Manipulation

#### Contingent Movements of Arms and Legs

Before being able to grasp an object and manipulate it—which infants start to do between 3 months and 5 months—they may use their legs or arms to produce interesting effects on their environment. Conjugate reinforcement studies with the legs (Rovee and Rovee, 1969), or with the arms (Watanabe and Taga, 2006) show that 3-month-olds move the leg or the arm, when it is connected to a mobile and makes the mobile move, more than when it is not connected.

#### Grasping and Manipulating Objects

After the post-natal period when pre-reaching can be observed only occasionally (Trevarthen, 1984a), infants learn to approach and grasp objects presented in front of them; this happens between 3 months and 5 months (White et al., 1964; von Hofsten, 1989; Thelen et al., 1993; Sgandurra et al., 2012). Reaching movements are at first rather unstable and indirect (von Hofsten and Rönnqvist, 1988; Konczak et al., 1995) but by 6–7 months of age infants gain enough control of the deceleration and hand opening in relation to object properties to be able to grasp objects even when the objects are not stabilized (Fetters and Todd, 1987; Fagard, 1998). Progress in bimanual coordination, first for symmetrical grasping movements (Fagard and Jacquet, 1996), then for role-differentiated movements (Fagard, 1998; Kimmerle et al., 2010) occur during the second half of the first year. Role-differentiated bimanual movements are essential for manipulating, exploring and playing with objects.

Once the infant is able to grasp objects in a broad array of situations, it begins manipulating them in various ways. The main action of the first 6 months is mouthing, but this behavior decreases over the next few months (McCall, 1974; Palmer, 1989). Over this period, more diverse behaviors are increasingly observed, such as swapping, banging, exploring visually while orienting; in addition, these exploratory behaviors become increasingly adapted to object properties (Ruff, 1984; Palmer, 1989; Fagard and Lockman, 2005; Schum et al., 2011). Infants seem particularly inclined to shake an object that makes a noise when shaken. They scratch an object with a ridged surface, wrinkle a piece of paper, bang on a table with a solid object, separate the two parts of a breakable object, etc. It seems as if infants are looking for the maximum effects they can get from the manipulation. Infants seem to enjoy new effects, but also to enjoy repeating known effects, as if to test their power of action on the object. At this stage of development, there is no doubt anymore about the playful character of the infant’s sensorimotor behavior.

#### Interacting With Others

Micro-analysis on observations of mother-infant interactions suggest that playful situations between mothers and infants emerge very early in life and play an important role in day-to-day interactions. Around 2- to 3 months infants begin to show contingent facial expressions (smiles) to the mother in situations of dyadic interactions (Trevarthen, 1984b). By 3 months, when infants become interested in other objects and mothers play with them in a triadic relation, mothers capture their infants’ attention by means of vocalizations, tactile stimulation and facial expressions, causing the first observed laughing expressions in infants. According to Bruner and Sherwood (1976), some of these routines later acquire a specific format such as the «peekaboo» game. Ever since Bruner’s first observations of this game, the «peekaboo» has been studied around the world and has been shown to be a universal means of interaction (Fernald and O’Neill, 1993). A recent study showed that infants as young as 4 months of age engage in peekaboo and take turns in the game, and that their participation increases at 6 months of age (Nomikou et al., 2017). The peekaboo game seems to be the most frequently observed playful behavior in infants and it has the key components of play, since it is repetitive, joyful for infants (smiling and laughing) and its only aim is pleasure. At 6 months, other playful behaviors seem to develop, including humorous behavior. For instance, infants start to actively manipulate other people by repeating actions that make other people laugh (Reddy, 2001; Mireault and Reddy, 2016). By the end of their first year, infants engage in new humorous actions to make other people laugh and by 18 months, they can discriminate between play contexts and other contexts such as lying or intentionally making mistakes (Hoicka and Gattis, 2008).

Taken together, these studies suggest that interacting develops from a very early age through repetitive and structured interactions.

## Conclusion

Fetal movements have often been interpreted as a way for the fetus to practice and exercise its emerging motor system, participating in its maturation. For instance, when pathology creates a relative absence of GMs, the limbs do not develop normally and show deformities (Piontelli, 2010). However, one has often underestimated other fundamental functions of GMs and isolated movements which makes them a direct precursor of infant’s exploratory sensorimotor behavior. GMs and isolated movements allow the fetus to explore its body as well as its environment, and to discover contingencies. In other words, fetal movements seem to already display the exploratory behavior observed in infants: curiosity and intrinsic motivation, detection of contingencies, repetition of “rewarding” actions (actions leading to an effect), intentionality and some motor control. This early capacity to detect contingencies, which the observation of goal-directed movements *in utero* suggests, may be a continually present mechanism determining the movements of infants after birth, despite the relative discontinuity at the moment of birth itself.

## Author Contributions

JF wrote the first draft of the article. All the other authors contributed equally to the final writing.

## Conflict of Interest Statement

The authors declare that the research was conducted in the absence of any commercial or financial relationships that could be construed as a potential conflict of interest.

## Footnotes

^1^Since the mid-late 1970s ultrasound recordings allows observing the fetal behavior for several minutes in a row. More recently, 4D ultrasound produces computerized reconstructions of the fetus in motion. Off-line analyses are made frame by frame, and any observable action or reaction, when the fetus is in an alert state, is coded. This leads to a classification of fetal movements largely based on Prechtl’s pioneering work.

^2^Fetal movements appear, increase in frequency, sometimes reach a plateau and decrease (in general). Not all authors agree on the precise timing of these changes in occurrences. The difference may be due to the quality of the ultrasound recording, the number of fetuses observed and the number of observations for each fetus, the variability of movements between and within fetuses, as well as to the length of time of each observation (15–60 min). However, the order of appearance of movements is quite similar from one author to the next.

^3^A model of light transmission from the external environment to the uterus seems to indicate that illumination of the cavity, although variable, allows for some visual experience before birth (Del Giudice, 2011).
